# Pyruvate Dehydrogenase Kinase 4 Deficiency Increases Tumorigenesis in a Murine Model of Bladder Cancer

**DOI:** 10.3390/cancers15061654

**Published:** 2023-03-08

**Authors:** Benjamin L. Woolbright, Ganeshkumar Rajendran, Erika Abbott, Austin Martin, Ryan Didde, Katie Dennis, Robert A. Harris, John A. Taylor

**Affiliations:** 1 Department of Urology, University of Kansas Medical Center, Kansas City, KS 66160, USA; 2 School of Medicine, Kansas University Medical Center, Kansas City, KS 66160, USA; 3 Department of Pathology, Kansas University Medical Center, Kansas City, KS 66160, USA; 4 Department of Biochemistry and Molecular Biology, Indiana University School of Medicine, Indianapolis, IN 46202, USA

**Keywords:** bladder cancer, mouse, PDK4, BBN, PDH

## Abstract

**Simple Summary:**

Pyruvate dehydrogenase kinase 4 (PDK4) is a protein that serves as a switch for how the body regulates metabolism. Prior research indicates that blocking the effect of PDK4 with some drugs slows the growth of bladder cancer cells. These experiments relied on cancer cell lines and not tumors grown in mouse models of cancer, which are more closely related to human disease. In a validated mouse model of bladder cancer, mice that did not express PDK4 were found to have larger tumors than mice expressing PDK4 at later points of tumor progression. As tumors became larger, there was a loss of expression of PDK4 in mice that normally expressed it. Human samples with bladder cancer had lower expression of PDK4 than those without bladder cancer. These data indicate that PDK4 may be an unexpected tumor suppressor in bladder cancer.

**Abstract:**

Pyruvate dehydrogenase kinase 4 (PDK4) is a mitochondrial isozyme in the PDK family (PDK1-4) partially responsible for phosphorylation of pyruvate dehydrogenase (PDH). Phosphorylation of PDH is thought to result in a pro-proliferative shift in metabolism that sustains growth of cancer cells. Previous data from our lab indicate the pan-PDK inhibitor dichloroacetate (DCA) or acute genetic knockdown of PDK4 blocks proliferation of bladder cancer (BCa) cells. The goal of this study was to determine the role of PDK4 in an in vivo BCa model, with the hypothesis that genetic depletion of PDK4 would impair formation of BCa. PDK4^−/−^ or WT animals were exposed to N-Butyl-N-(4-hydroxybutyl) nitrosamine (BBN) for 16 weeks, and tumors were allowed to develop for up to 7 additional weeks. PDK4^−/−^ mice had significantly larger tumors at later time points. When animals were treated with cisplatin, PDK4^−/−^ animals still had larger tumors than WT mice. PDK4 expression was assessed in human tissue and in mice. WT mice lost expression of PDK4 as tumors became muscle-invasive. Similar results were observed in human samples, wherein tumors had less expression of PDK4 than benign tissue. In summary, PDK4 has a complex, multifunctional role in BCa and may represent an underrecognized tumor suppressor.

## 1. Introduction

Bladder cancer (BCa) is a highly morbid disease, particularly in advanced stages [1,2]. Standard-of-care treatment for muscle-invasive disease remains surgical intervention, with added benefit provided by neoadjuvant cisplatin-based chemotherapy [2,3]. Biomarker studies have begun to define which populations of BCa patient might be most likely to benefit from cisplatin-based therapy, but a significant portion of BCa patients receive no benefit currently [4,5]. Novel immunotherapies targeting PD-1:PD-L1 interactions have been approved in BCa and provide important systemic treatment options [2]. However, not all patients respond to immunotherapy, and new therapeutic adjuvants will likely be needed to improve overall response [6]. Currently, there remains a large gap in treatment efficacy for many patients with BCa, particularly those that are cisplatin-ineligible. Adjuvant treatments that improve cisplatin or immune-based therapy and novel treatments are sorely needed [1,2].

Pyruvate dehydrogenase kinase 4 (PDK4) is a mitochondrial enzyme responsible for phosphorylation of pyruvate dehydrogenase (PDH) and one of the four pyruvate dehydrogenase kinase isozymes, PDK1-4 [7,8,9]. PDH converts pyruvate to acetyl-CoA and serves as a major hub in many metabolic processes necessary to generate the metabolic components required for cellular proliferation. Phosphorylation of PDH prevents the conversion of pyruvate to acetyl-CoA [9,10,11]. The inhibition of PDH increases intracellular levels of pyruvate, which cancer cells can then convert to lactate via lactate dehydrogenase to recycle NAD^+^ and produce ATP via aerobic glycolysis, which can enhance growth rates [9,10,11]. This process has long been of interest to the cancer field, but more recent data indicate that this process likely benefits cells in multiple ways, including through the production of oxidative biomass, in addition to the production of ATP, as the mitochondria of cancer cells are metabolically very active [12,13,14,15]. Inhibition of PDKs as a therapeutic target has been pursued in many different cancers as a means of slowing cancer growth [9,16,17,18,19,20]. The most commonly used inhibitor is dichloroacetate (DCA), a pan-inhibitor of all four PDKs. DCA requires high drug concentrations to be effective in vitro due to its low potency but reduces tumor growth in multiple cancers, including breast, kidney, lung, and liver cancers [7]. DCA has some affinity for all four PDKs, which means it minimally allows for differentiation of the roles of individual PDK enzymes. Other more specific inhibitors that target the PDK:PDH axis, including AZD7545, CPI-613, and VER-246608, have also proven effective against cancer in multiple cell lines [9]. As such, inhibition of PDKs has consistently been proposed as a therapeutic target in cancer.

Recent evidence suggests that PDK4 inhibition may be more complicated than inhibitor studies first indicated. Genetic knockout of PDK4 in hepatocellular carcinoma (HCC) cells resulted in increased proliferation [21,22]. Similarly, PDK4-deficient non-small cell lung carcinoma (NSCLC) cells grew more aggressively, which was proposed to occur through altered regulation of lipid metabolism [23]. Prior data from our lab indicate that BCa cells are responsive to pan-inhibition of PDKs with DCA and that treatment also enhances the effect of cisplatin. Moreover, acute knockdown of PDK4 with siRNA slows BCa growth rates [24]. However, there is currently no understanding of the effect of PDK4 deficiency in orthotopic models of BCa. Given the observed effect of DCA and cisplatin in BCa cell lines, we sought to understand how PDK4-deficient mice would respond to challenge with a BCa-specific carcinogen, N-Butyl-N-(4-hydroxybutyl)nitrosamine (BBN) [25,26]. We hypothesized that PDK4 deficiency would reduce tumor formation and enhance cisplatin efficacy based on these data.

## 2. Methods and Materials

### 2.1. Mice

PDK4^−/−^ animals were a kind gift from Dr. Lisa Zhang and originally produced in the lab of Dr. Robert Harris. PDK4^−/−^ animals were bred homozygously in house, and male mice were used for experimentation due to innate resistance to BBN in female animals. C57BL/6 controls were acquired from Jackson Labs. All experiments were approved by the Kansas University Medical Center IACUC prior to onset of experimentation.

### 2.2. BBN Protocol

Animals were placed on 0.05% N-Butyl-N-(4-hydroxybutyl)nitrosamine (BBN) (TCI America, Portland, OR) in drinking water and provided food and water containing BBN *ad libitum* for 16 weeks to initiate tumorigenesis. Water containing BBN was provided in opaque bottles to reduce degradation. Cisplatin was dissolved in normal saline via heating at 37 °C for ~5–10 min with mild shaking intermittently until fully dissolved. For experiments with WT and PDK4^−/−^ mice, cisplatin was administered twice weekly at 3 mg/kg i.p. for 3 weeks in total. To assess tumor formation, bladders were bisected along the mid-sagittal plane and then weighed. After weighing, bladders were placed in neutral buffered formalin for future immunohistochemistry. One animal in the 23 w group (WT) was found moribund four days prior to the end of the study but was included in the study due to obvious tumor formation. Chemicals were acquired from Sigma (St. Louis, MO, USA) unless otherwise noted.

### 2.3. Immunohistochemistry

Tissue was formalin-fixed and then embedded in paraffin. Then, 5 µM sections were cut for analysis. Endogenous peroxidase was quenched with a peroxidase suppressor (Thermo, Waltham, MA, USA). Antigen retrieval was performed by submerging tissue in a boiling citrate buffer (pH6) for 10 m. Immunohistochemistry was performed with a Vectastain Elite ABC kit (Vector Labs, Burlingame, CA, USA) as per the manufacturer’s protocol, followed by visualization with 3′,3′-diaminobenzidine. Ki67 antibody was acquired from Cell Signaling (clone D3B5), and PDK4 antibody was acquired from Sigma (HPA056731). Ki67-positive cells were independently quantified in three to four separate fields from each mouse by two separate scientists and then averaged. The H index in human tumors was calculated by evaluating the percentage and degree of positivity in epithelial sections to reduce variability and account for the relatively low degree of epithelium in bladder tissue samples. Any degree of nuclear positivity was considered positive. Terminal deoxynucleotidyl transferase dUTP nick end labeling (TUNEL) staining was performed using a Roche in situ cell death detection kit (Roche, Basel, Switzerland). Microscopy was performed on an Olympus IX73 (Tokyo, Japan) using cellSens Standard software version 4.1. H&E staining was carried out using standard methodology. Briefly, tissue was deparaffinized, rehydrated, exposed to hematoxylin and eosin, and serially dehydrated in alcohol and xylene before mounting. Tumor stage was evaluated by a board-certified genitourinary pathologist (KD).

### 2.4. Human Tissue and TCGA Analysis

Data were acquired from either the University of Alabama at Birmingham Cancer data analysis portal (UALCAN), Gene Expression Profiling Interactive Analysis (GEPIA), or Xenabrowser [27,28,29,30]. Xenabrowser was used to access values for high-grade samples, in addition to the small number of incidentally acquired low-grade samples in the database, and values for solid normal tissue samples were included in the database as adjacent controls. Samples for which there were no values or no establishment of grade on Xenabrowser were omitted from this analysis. UALCAN and GEPIA were also used to assess normal versus cancer samples. Small differences in overall group numbers are reflective of differences inherent to the way each program analyzes TCGA data. Solid normal tissue represents adjacent solid tissue and not necessarily truly normal tissue. The BCa tumor microarray was acquired commercially from US BIOMAX (Derwood, MA, USA) and represented tumors of varied stage and grade, in additional to normal controls.

### 2.5. qPCR

RNA was isolated using Trizol. QPCR was carried out using TaqMan-based primers and expressed using the delta–delta CT method.

### 2.6. Statistics

Statistics were performed using Sigmaplot. Data were evaluated for normality and then compared via *t*-test or by either Kruskal Wallis or Mann–Whitney test with post hoc correction. * *p* < 0.05. Data are expressed as mean +/− standard error.

## 3. Results

### 3.1. PDK4^−/−^ Animals Have Larger, Higher-Stage Tumors after BBN Treatment at Later Time Points of Tumor Formation

**Figure 1:**
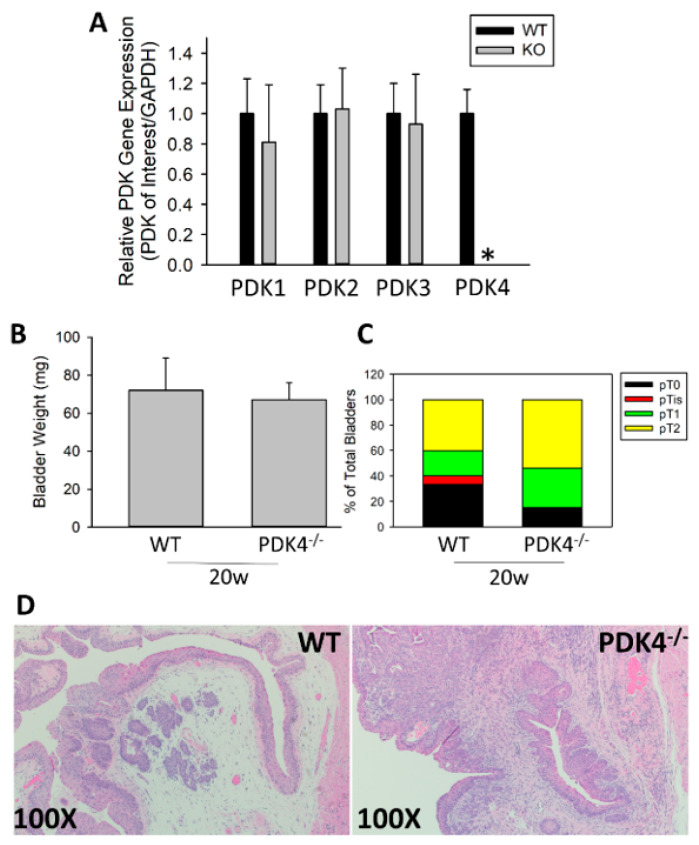
Early tumors are similar between WT and PDK4^−/−^ mice after BBN treatment. PDK4 gene expression was determined at baseline in bladders of untreated animals (**A**) WT (*n* = 15) or PDK4^−/−^ (*n* = 13) animals were placed on BBN for 16 w, followed by sacrifice at 4 w post BBN (20 w total). Bladder weights (**B**) and tumor stage (**C**,**D**) were measured. * *p* < 0.05.

To evaluate the role of PDK4 in BCa development, the BBN-induced BCa model was used. This model has been shown to genetically and phenotypically recapitulate the basal subtype of human BCa and represents a gold-standard model in the field [31,32]. We initially assessed levels of the PDK isozymes in the bladder of PDK4^−/−^ animals to determine whether PDK4 was selectively reduced in PDK4^−/−^ animals. PDK1-3 expression was unchanged in the bladder, whereas PDK4 transcripts were largely undetectable (Figure 1A). To determine how PDK4^−/−^ animals respond to BBN-induced BCa, WT or PDK4^−/−^ mice were treated with 0.05% BBN via drinking water for 16 weeks, followed by 4 additional weeks with normal water (no BBN) for continued tumor formation (20 w total). No difference in tumor weight between WT and PDK4^−/−^ mice was observed after 4 additional weeks of tumor formation time (Figure 1B). Tumor staging via H&E staining was performed by a board-certified pathologist, but again, no difference was observed between WT and PDK4^−/−^ animals (Figure 1C,D). These data indicate that WT and PDK4^−/−^ animals did not demonstrate any difference in early tumor formation.

**Figure 2:**
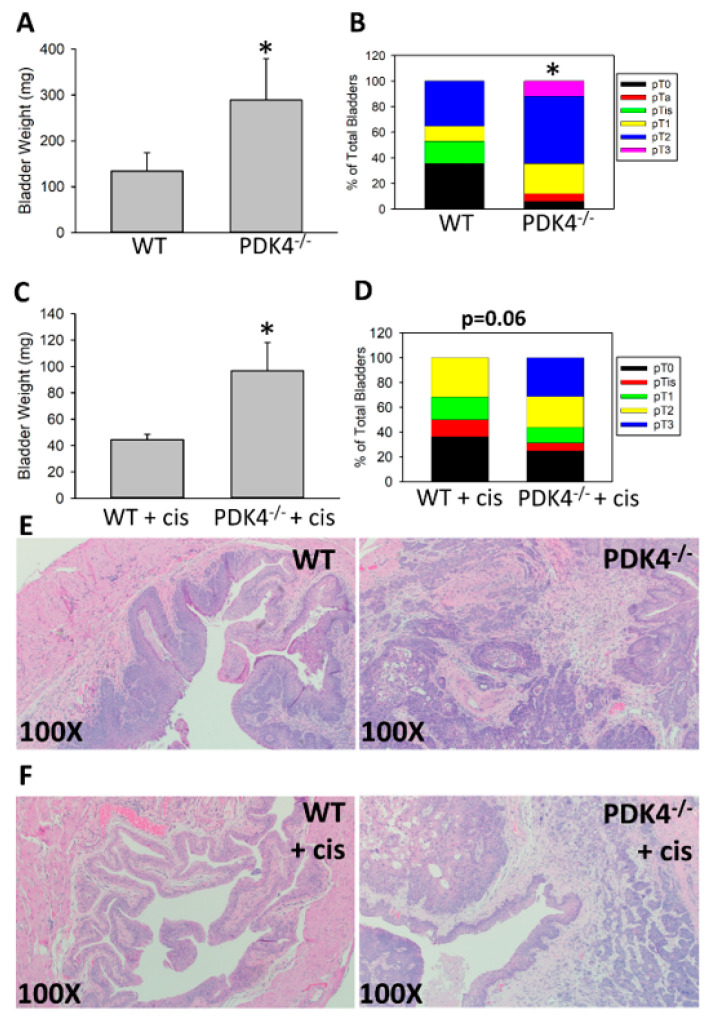
PDK4^−/−^ mice have larger BBN-induced tumors than WT mice at later tumor stages with and without cisplatin treatment. WT (*n* = 17) or PDK4^−/−^ (*n* = 17) animals were placed on BBN for 16 w, and tumors were allowed to grow for another 7 w. Bladder weights (**A**) and tumor stage (**B**) were assessed. In a separate experiment using WT (*n* = 22) and PDK4^−/−^ (*n* = 16) mice, cisplatin was administered twice weekly at 3 mg/kg for the final 3 weeks. Bladder weights (**C**) and tumor stage (**D**) were measured. Representative H&E images are presented in (**E**,**F**) for untreated and cisplatin-treated animals, respectively. * *p* < 0.05.

We next sought to determine whether PDK4 deficiency would alter late-stage tumor formation or progression. Moreover, prior data from our lab indicated that PDK4 inhibition may improve cisplatin-based therapy. To assess this, we again initiated tumor formation with 0.05% BBN for 16 w and assessed tumor levels after 7 additional weeks with or without the presence of 3 mg/kg cisplatin twice weekly in the last 3 weeks. PDK4^−/−^ animals had larger tumors than their WT counterparts after 7 w of additional tumor growth as assessed by bladder weights, which is a surrogate for tumor volume (23 w in total) (Figure 2A). Tumor staging based on H&E staining yielded a significant increase in tumor stage in PDK4^−/−^ animals compared to WT controls (Figure 2B,E). In animals treated with cisplatin, similar results were obtained; PDK4^−/−^ animals, again, had larger tumors than WT controls as assessed by bladder weight (Figure 2C). However, in contrast, no overall difference in tumor stage was observed in cisplatin-treated animals (Figure 2D,F), although the PDK4^−/−^ group had persistent T3 tumors that were absent in the WT group. Overall, these data support the idea that PDK4 acts as an antitumorigenic agent in late-stage tumors but not early-stage tumors.

### 3.2. Increased Proliferative Potential of PDK4^−/−^ Tumors

**Figure 3:**
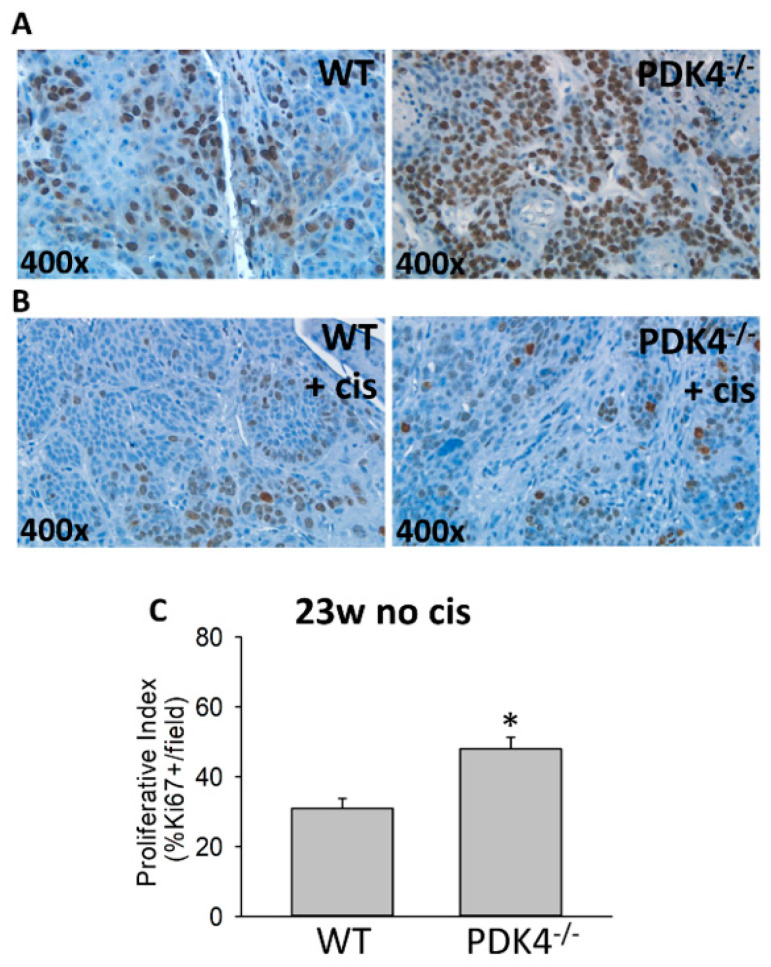
Increased proliferation in PDK4^−/−^ tumors compared to controls. WT and PDK4^−/−^ tumors were assessed for Ki67 positivity via immunohistochemistry. Untreated (**A**) and cisplatin-treated (**B**) tumors are presented at the indicated magnification. Proliferative index (**C**) was assessed by evaluating Ki67 positivity in three to four separate fields from each mouse. * *p* < 0.05.

To assess why PDK4^−/−^ tumors were larger, we initially hypothesized that PDK4^−/−^ tumors may be resistant to apoptosis due to altered metabolism, leading to larger tumor size due to a reduction in cellular turnover. To assess this, TUNEL^+^ cells were quantified in WT and PDK4^−/−^ animals; however, the overall rate of TUNEL^+^ cells was relatively low in both WT and PDK4^−/−^ animals, suggesting that this was an unlikely mechanism (Supplementary Figure S1). We next hypothesized that PDK4^−/−^ might lead to a more proliferative phenotype. Proliferation was assessed by immunohistochemistry for Ki67 (Figure 3A,B). Ki67^+^ cell numbers were higher in PDK4^−/−^ tumors, although not in those treated with cisplatin (Figure 3B,C). Ki67^+^ cells remained in both WT and PDK4^−/−^ animals, suggesting the continued presence of proliferating cells after treatment with cisplatin. The increase in Ki67^+^ cells indicates that PDK4^−/−^ tumors were more proliferative.

### 3.3. PDK4 Expression Is Suppressed in Murine and Human BCa

**Figure 4:**
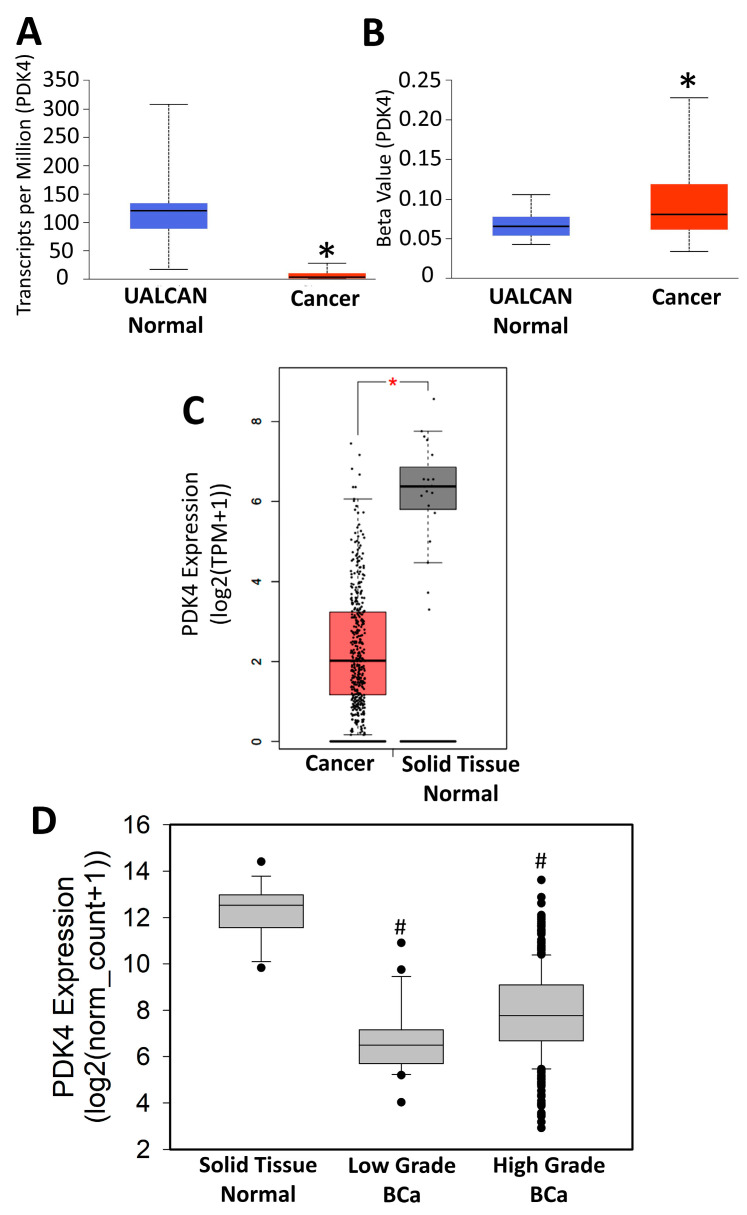
PDK4 gene expression in TCGA. PDK4 gene expression (**A**) (normal *n* = 19, tumor *n* = 408)and promoter methylation values (**B**) (normal n = 21, tumor n = 418) acquired via UALCAN. PDK4 gene expression derived from TCGA using GEPIA (**C**) (normal, *n* = 19; tumor, *n* = 404) or Xenabrowser (**D**) (normal, *n* = 19; low grade, *n* = 21; high grade, *n* = 383). * *p* < 0.05 versus solid normal tissue # *p* < 0.05 versus all other samples.

**Figure 5:**
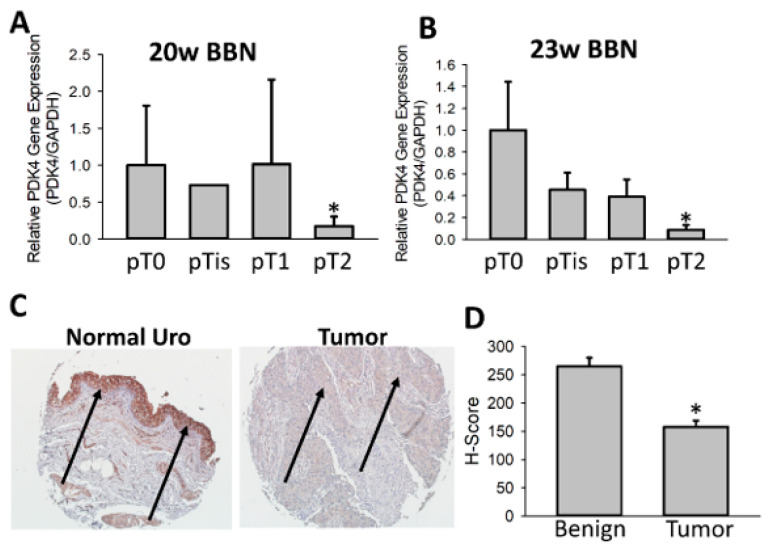
PDK4 is suppressed during BCa tumorigenesis. PDK4 gene expression was measured in RNA derived from WT murine tumors exposed to BBN. Tumors were delineated by stage by a board-certified pathologist to assess PDK4 expression across stages (**A**,**B**). A human tumor microarray was stained for PDK4 using normal (*n* = 18) or tumor samples (*n* = 77) (**C**). Tumors were quantified using H score (**D**). Arrows indicate urothelium/tumor. * *p* < 0.05.

To further understand why PDK4^−/−^ tumors were larger and more proliferative, we next assessed the expression patterns of PDK4 in murine and human tumors. Our lab previously published data demonstrating a significant increase in PDK4 between low-grade and high-grade samples [24]. However, analysis of BCa specimens in TCGA demonstrated a novel trend, as PDK4 expression was suppressed in cancer compared to normal controls (Figure 4A). This was consistent with increased methylation of the PDK4 promoter in TCGA tumor specimens compared to controls, suggesting a potential epigenetic mechanism of PDK4 suppression (Figure 4B). These data were confirmed using the GEPIA database (Figure 4C) [29]. GEPIA analysis including Genotype-Tissue Expression (GTEx)-provided normal bladder tissue further confirmed our findings (data accessed via http://gepia.cancer-pku.cn/index.html, accessed on 25 January 2023). To better understand this difference, TCGA was used to evaluate PDK4 expression in a small group of low-grade tumors that were defined in the cohort. While both low-grade and high-grade cancers exhibited reductions in PDK4, low-grade tumors had a greater reduction than high-grade tumors (Figure 4D). To confirm these data in our mice, we evaluated PDK4 expression in WT animals. Notably, the BBN model does not produce low-grade tumors, and therefore, the only analysis possible was to understand differences in PDK4 expression in BBN-exposed animals with high-grade tumors versus animals exposed to BBN but without tumors. Animals with muscle-invasive (pT2+) tumors consistently had lower expression of PDK4 than animals exposed to BBN but with benign tissue (Figure 5A,B). Murine antibodies for PDK4 were not suitable for IHC, so instead, PDK4 was stained in a human tumor microarray. While the urothelium in normal tissue stained positive for PDK4, it was largely absent in the majority of tumor specimens (Figure 5C) Scoring tumors via H index demonstrated a statistically significant reduction in PDK4 expression in the epithelium compared to WT controls (Figure 5D). These data indicate that PDK4 is largely lost in more advanced tumors in both mice and humans.

In totality, these data indicate that PDK4 likely plays a highly complex role with differential regulation across the spectrum of BCa tumors. Experiments with chronic genetic loss of PDK4 indicate that it may have tumor-suppressive functions, whereas, acute inhibition of all PDKs with agents such as DCA yields a therapeutic effect based on prior experiments [24]. More studies are needed to better elucidate the role of PDK4 and PDKs generally in BCa.

## 4. Discussion

The mitochondrial isozymes PDKs 1-4 are responsible for phosphorylation of PDH and serve as a tightly controlled biological switch to manage cellular energy needs. When PDH is phosphorylated by PDKs, pyruvate is instead metabolized by other enzymes such as lactate dehydrogenase, which produces lactate (aerobic glycolysis), and pyruvate carboxylase, which produces oxaloacetate [7,33]. The original Warburg hypothesis suggested that the production of ATP by aerobic glycolysis was a major reason why cancer cells were capable of unlimited proliferation; however, recent data suggest that cancer cells undergo a broad cellular reprogramming of metabolism that allows access to many different metabolically favorable reactions for production of otherwise rate-limiting materials [12,13,14]. Inhibition of PDKs has widely been proposed as a means of slowing cancer proliferation by promoting pyruvate oxidation, which shifts metabolism away from aerobic glycolysis and towards mitochondrial oxidative metabolism [9,14,34]. This both limits production of biomass needed for proliferation and enhances production of mitochondrial ROS, which can be toxic to the cell. Supporting this idea, numerous cancer cell lines are sensitive to PDK inhibition [7,9]. We and others have found that pan-inhibition of all PDKs with DCA can block cellular proliferation. This has alternately been attributed to knockdown of single PDK enzymes or, at times, knockdown of all PDK isozymes [7]. In our own studies and others, acute knockdown of PDK4 with siRNA has been found to limit cellular proliferation in BCa cell lines, although the current study indicates that the biology is likely far more complex in vivo [24,35]. Using the BBN mouse model, we found that PDK4^−/−^ animals had larger bladder tumors than their counterparts and that PDK4 itself is largely lost during tumor formation in both humans and mice. While animals exposed to cisplatin did not have higher stage tumors, they still had larger bladders than their WT counterparts, further confirming the possibility that PDK4^−/−^ animals are more predisposed to tumor progression. The totality of these data indicate that PDK4 specifically may function as a tumor suppressor in BCa, particularly in later-stage tumors. Moreover, our data indicate that the role of PDK4 is likely more complex in vivo than BCa cell lines can accurately model; therefore, understanding the role of PDK4 in BCa will require complicated murine models.

Our initial hypothesis was that PDK4^−/−^ animals would exhibit reduced tumor formation and be more responsive to cisplatin based on prior in vitro data in BCa, in addition to numerous studies demonstrating an antitumorigenic effect of PDK inhibition [7,20,24,34]. The primary outcome of this paper is the surprising finding that PDK4^−/−^ animals had larger tumors, which is indicative of an antitumorigenic effect of PDK4 (Figure 2 and Figure 3). PDK4^−/−^ tumors had higher amounts of Ki67^+^ cells, which is indicative of a more proliferative phenotype. The underlying mechanism was not defined in this study. Prior work indicated that knockout of PDK4 can cause pro-proliferative changes in tumor cells by enhancing lipogenesis in other cancers [21,22,23]. Knockdown of PDK4 promotes growth of A549 and NCI-H1299 lung cancer cells, whereas overexpression of miR-182 reduces tumor growth via downregulation of PDK4 [23]. This has been proposed to be a result of downstream actions on lipogenesis in tissues with high lipogenic activity [23,36]. Similarly, knockdown of PDK4 increases lipogenesis and has been reported to increase HCC growth rates, with similar findings in prostate cancer [21,22,36]. The dependency/capacity of each tumor on de novo fatty synthesis may be a predictor of whether or not PDK4 acts as a tumor suppressor, although additional studies are needed in this area across a broader range of tumors [36]. As current research indicates that ATP production is not the likely limiting factor for cancer proliferation, the action of PDK4 on metabolism may ultimately reduce metabolic intermediates necessary for proliferation. This study was not able to define an exact mechanism as to why PDK4^−/−^ tumors were larger or were larger exclusively at later time point, but given that BBN-induced tumors increase in stage over time, one possibility is that PDK4 constrains tumors that reach a certain stage due to underlying cellular signaling. Knockout of this protein then alleviates this constraint and allows tumors to grow more quickly. Cell lines do not accurately recapitulate tumor stage or the exact underlying microenvironment; therefore, murine experiments are needed to understand how this occurs. Selectively depleting or overexpressing PDK4 at specific points during tumor formation in mice may allow for a better understanding of how and when PDK4 yields its effects. Because cell line data and murine data on the role of PDK4 have been somewhat discordant in both BCa and other tumors, the most effective way to accomplish this may require the use of conditional knockout animals using a Cre/lox system to selectively deplete PDK4 at different points in tumor formation. This could be further honed to different cellular populations to provide a robust and granular explanation as to which cells are most effected by loss of PDK4 and how this promotes tumor formation.

One area that we did not fully explore mechanistically is the role of PDK4 in tumor inflammation. PDKs can alter metabolism in T cells as in other cell lines. The loss of other PDKs such as PDK1 can control T-cell fate by altering cellular metabolism [37]. While BBN induces some degree of inflammation by itself, inflammation is dramatically increased when a tumor is present [38]. A possible hypothesis is that PDK4 alters the ability of T cells to robustly respond to tumors. Depletion of both PDK2 and PDK4 but not PDK4 alone prevents the conversion of macrophages to the M1 phenotype in other models. If the loss of PDK4 alone is capable of pushing macrophages towards the M2 phenotype in this model, it could be another potential mechanism of reduced immunity [39]. While these studies are outside the scope of this work, understanding how PDK4 loss affects T cells and other immune populations may shed light on why PDK4^−/−^ animals have larger tumors in immunologically intact in vivo models despite opposite result in cell lines.

Non-canonical actions of PDK4 have recently been reported [35]. Multiple proteins other than PDH have been identified as potential substrates of PDK4 [35,40]. This includes a partially defined signaling axis between PDK4-Septin2 (SEPT2)-dynamin-related protein 1 (DRP1), which regulates mitochondrial fission [40]. Altered mitochondrial fission/fusion dynamics have been demonstrated in multiple cancers, with some reports indicating that alteration of fission/fusion may be therapeutically useful [41,42]. If loss of PDK4 prevents normal mitochondrial fission, this may constrain tumor growth by increasing mitochondrial instability, thereby limiting cell proliferation. Because mitochondrial fusion and fission processes are critical to normal biology, the ability to alter/modulate DRP1 activity indirectly through related proteins in the signaling pathway may be therapeutically viable, and therefore understanding the role of PDK4 in fission may be useful in the near future [41]. Currently understood activity of PDK4 on PDH, in addition to inhibitor studies, suggests that PDK4 should function as a positive regulator of tumor formation. The advent of numerous PDK4 client substrates offers the possibility that a significant biological activity remains under investigation and may be highly relevant to future studies, particularly in regard to the ability of PDK4 to suppress tumor formation rather than enhance it. Mechanistic studies investigating the effects of PDK4 on these proteins are needed to better understand how PDK4 suppresses tumor formation.

PDK4 expression was lost in muscle-invasive BCa tumors in murine studies and was consistently repressed in BCa tumors compared to solid normal tissue in analysis of human samples (Figure 4 and Figure 5). In a limited set of human samples present in the TCGA database, PDK4 expression was markedly lower in low-grade tumors compared to high-grade tumors. This is notable because we found that the tumor suppressive effects of PDK4 are most noticeable in later stages of tumor formation in mice (Figure 2). BCa tumors progress through either the CIS muscle-invasive pathway of development or the low-grade pathway of development according to recent literature [43,44]. The BBN model does not produce low-grade tumors, which prohibits analysis of how PDK4 expression in mice alters the low-grade pathway. Because we observed a marked reduction in PDK4 in low-grade samples, PDK4 may also be highly relevant in this pathway, although this would require a separate model to assess. Because BBN is highly proinflammatory and alters the tumor microenvironment, the observed reduction in PDK4 RNA expression in mice may ultimately be related to the presence of inflammatory cells that alter the overall RNA expression profile of the tumor; however, the combination of human and mouse data indicates that the most likely answer remains the direct suppression of PDK4 expression in BCa tumors. TCGA analysis indicates a reciprocal change in PDK4 expression/promoter methylation indicative of an epigenetic silencing mechanism in BCa (Figure 4). Similar observations have been made in HCC and are supported by laboratory studies indicating that arsenic can methylate and reduce the expression of PDK4 [45,46]. Epigenetic silencing of PDK4 has also been reported in other cancers [47]. In addition to epigenetic changes, PDK4 can also be regulated by miRs and undergoes transcriptional regulation in response to many biological stimuli; therefore, multiple mechanisms are plausible [23]. Importantly, through the use of a tumor microarray, we were able to show that PDK4 protein is also reduced in BCa tissue compared to control tissue from a commercially available tumor microarray using human samples. The exact mechanism of this action is undetermined and may involve multiple factors, although given the consistent data around epigenetic suppression, this is a likely contributing factor. Functionally, human studies on HCC indicate that loss of PDK4 is associated with more advanced tumors and worse outcomes, supporting the idea that PDK4 may function as a tumor suppressor [48]. Future studies should delineate what is the precipitating factor for loss of PDK4 that occurs during tumor formation and whether this is functionally related to more aggressive tumor outcomes.

The observation that PDK4 was downregulated in BBN tumors is a surprising finding, given prior in vitro and in vivo results using low-grade and high-grade tumors [24]. The data from our BBN model represent novel findings; however, we were able to corroborate the prior observed changes in low- vs. high-grade samples. The novel finding here is the comparison to solid normal tissue, wherein both late-stage murine tumors and human tumors had reduced PDK4 expression. Our findings are also corroborated by other studies. Fantini et al. previously found that PDK4 was suppressed in established BBN tumors but not animals administered BBN for a briefer period [32]. This supports the idea that the loss of PDK4 is associated with tumor formation and not just the inflammatory phase of BBN or the precancerous phase. The mechanisms that dictate the loss of PDK4 during cancer formation need to be fully evaluated in the context of both a wide range of human samples and in mice that allow for conditional or temporal knockout.

## 5. Conclusions

In conclusion, we report the novel finding that PDK4 may exhibit tumor-suppressor activity in BCa, which suggests a far more complex role for PDK4 during BCa carcinogenesis than previously described. Given the recent identification of alternate substrates of PDK4, understanding how it affects tumor formation both in the context of PDH and in the context of its other substrates is imperative. Future work will aim to understand how both canonical and non-canonical PDK4 activity reduces tumor growth and progression.

## Data Availability

All reasonable requests for data will be honored by the authors.

## Supplementary Materials

The following supporting information can be downloaded at: https://www.mdpi.com/article/10.3390/cancers15061654/s1, Figure S1: Immunohistology changes in WT and PDK4^−/−^ animals. Representative TUNEL staining in WT and PDK4^−/−^ animals.
