# Supplementary material for: Pyruvate Dehydrogenase Kinase 4 Deficiency Increases Tumorigenesis in a Murine Model of Bladder Cancer
Source: Cancers (Basel). 2023 Mar 8;15(6):1654. doi: 10.3390/cancers15061654 (PMC10046149; doi:10.3390/cancers15061654)
Supplement: Supplementary file 1 [file cancers-15-01654-s001.zip › cancers-2210773-supplementary.pdf]

S1

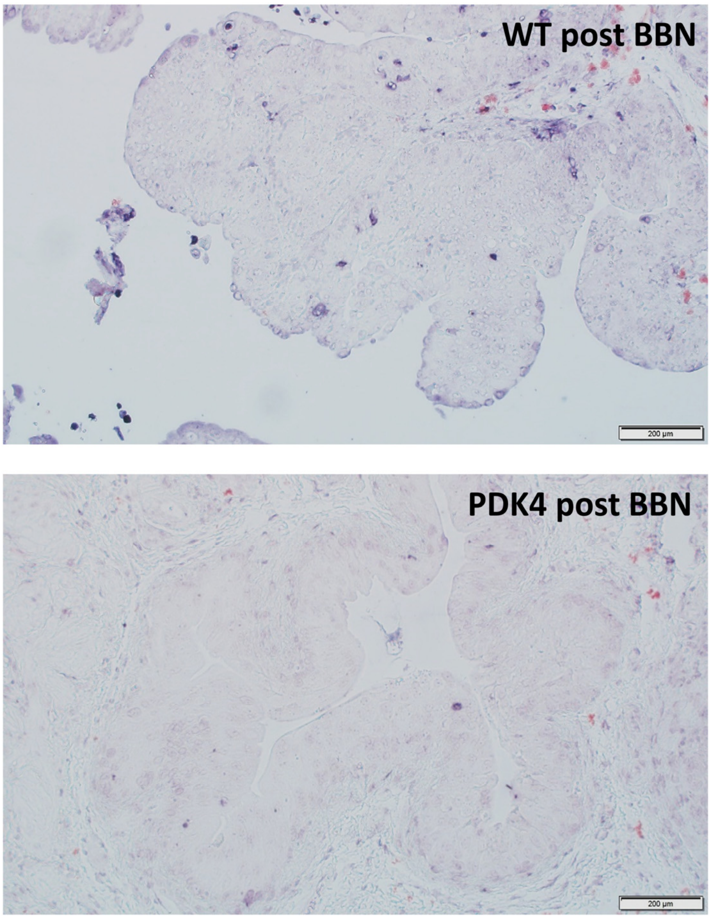

Supplementary Figure S1: Immunohistology changes in WT and PDK4$^{-/-}$ animals.

Representative TUNEL staining in WT and PDK4$^{-/-}$ animals.
